# Quality of Life of People with Mental Health Challenges and Problematic Substance Use while Engaged with an Exercise Physiology Service

**DOI:** 10.1007/s10597-025-01518-0

**Published:** 2025-09-18

**Authors:** Jane Kugelman, Meg Doohan, Brett Dyer, Jake O’Brien, Mridula Kayal, Justin Chapman

**Affiliations:** 1 School of Public Health, University of Queensland, Brisbane, Australia; 2 Centre for Mental Health, School of Pharmacy and Medical Sciences, Griffith University, Brisbane, Australia; 3 Griffith Biostatistics Unit, Griffith University, Brisbane, Australia; 4 Department of Gastroenterology and Hepatology, Metro South Health, Brisbane, Australia; 5 Mental Health Alcohol Tobacco and Other Drugs Service, Cairns and Hinterland Hospital and Health Service, Brisbane, Australia; 6 Addiction and Mental Health Service, Metro South Health, Brisbane, Australia

**Keywords:** Physical activity, Mental illness, Implementation, Exercise intervention, Multidisciplinary care, Community mental health

## Abstract

People with mental health challenges and problematic substance use may experience low quality of life (QoL) because of illness-related factors and psychosocial stressors. Evidence indicates that regular exercise can improve QoL for people with a range of mental health and substance use issues, and Accredited Exercise Physiologists (AEPs) provide evidence-based exercise interventions for people with a range of health conditions. This study examined QoL changes in people with mental health challenges and problematic substance use while engaged in an AEP service. The service was implemented over three years and involved weekly group exercise sessions and health education. Linear mixed-effects models were used to evaluate trajectories of QoL total score and each dimension (independent living, pain, senses, relationships, mental health, coping). A total of 295 participants were inducted, with 161 completing at least two assessments. QoL improved by 5.8% per year (95%CI: 1.8–9.8%), predominantly in mental health and coping dimensions. This study demonstrates the feasibility and adoption of AEP services to inform service planning for improving QoL in people with mental health challenges and problematic substance use.

**Supplementary Information:**

The online version contains supplementary material available at 10.1007/s10597-025-01518-0.

## Introduction

Mental health challenges and problematic substance use are the leading cause of disability globally (Whiteford et al., 2013). These include ‘common mental disorders’ such as depression and anxiety, alcohol and other drug disorders, and severe mental illnesses (SMIs) such as major depression, schizophrenia and bipolar disorders. In Australia, more than one in five adults aged between 16–85 years have experienced a common mental disorder and/or substance use disorder over the previous 12 months (Australian Bureau of Statistics, 2023), and the annual prevalence of SMIs is estimated to be around 1%. Disadvantage and psychosocial adversity associated with these conditions significantly reduce quality of life (QoL) (Evans et al., 2007), exacerbate distress, negatively impact self-esteem and self-confidence, and lead to a sense of hopelessness and demoralisation (Connell et al., 2012).

Exercise interventions can improve QoL in people with a range of mental health challenges or problematic substance use (Solmi et al., 2025; Vancampfort et al., 2025). Exercise interventions can also improve physical functioning and metabolic health in people with severe mental illnesses (Korman et al., 2023). Despite being an evidence-based treatment, people with mental health challenges and problematic substance use often face significant barriers to engaging in regular physical activity (Firth et al., 2016), which can be compounded by psychological distress (Chapman et al., 2016). Exercise programs must consider participants’ needs and values to maximise participation and support person-centred recovery (Tweed et al., 2021). People with mental illness commonly report preferences for individualised support provided by an exercise professional, affordable, and with a social component (Chapman et al., 2016). Qualitative studies have shown that people with mental illnesses value the social connection and sense of belonging resulting from participation in group-based exercise and lifestyle programs (Lund et al., 2019; Whybird et al., 2022). Exercise programs that foster autonomy, sense of belonging, and engagement in meaningful and enjoyable activities are needed to support recovery in people experiencing mental health challenges and problematic substance use.

Accredited Exercise Physiologists (AEPs) are tertiary-qualified professionals specialising in exercise therapy for people with complex conditions (Exercise & Sports Science Australia, 2024). Exercise interventions delivered by qualified exercise professionals may be more effective than those delivered by mental health staff or facilitators without exercise qualifications (Vancampfort et al., 2016). While AEPs are well-positioned to deliver evidence-based exercise interventions for people with mental and substance use disorders, there are few evaluated and published AEP service models to inform implementation in this area. Available models have demonstrated good feasibility (Fibbins et al., 2021; Furzer et al., 2021; Korman et al., 2020), acceptability (Furzer et al., 2021; Korman et al., 2020), and positive outcomes across a range of consumer care settings (Seymour et al., 2021; Teasdale et al., 2024). Global recommendations for implementing exercise programs in mental healthcare recommend integrating AEPs into mental healthcare, individual tailoring of exercise programming, involving participants’ social supports, and offering resources to support lifestyle changes (Teasdale, 2025), Further evidence on the impact of AEP-led interventions on QoL in people with mental health challenges or problematic substance use is needed to inform policy and practice in different contexts and settings.

The primary aim of this study was to evaluate outcomes of people with mental health challenges or problematic substance use while engaged with an AEP-led exercise and healthy lifestyle service. Objectives were to: (1) report procedural statistics of service provision; (2) evaluate the trajectory of QoL for participants after entering the service; and (3) examine the influence of attendance and participant characteristics on QoL trajectory. As a real-world evaluation, the analyses were exploratory with the intent of being hypothesis-generating rather than hypothesis testing, therefore there was no a priori hypothesis.

## Methods

This was a retrospective evaluation of an AEP-led community-based exercise and healthy lifestyle service for people experiencing mental health challenges or problematic substance use. The service was iteratively developed by a peer worker over 10 years (from 2010 to 2020; an overview of learnings is provided in Supplementary information) and delivered by the Queensland Police Citizen’s Youth Welfare Association (PCYC Queensland) in partnership with mental health organisations and services. The current evaluation was conducted over a three-year period (October 2020 to October 2023). Ethical approval was obtained from QIMR Berghofer Medical Research Human Research Ethics Committee (P2398).

Individuals were referred to the service from general practice, private clinics, non-government organisations, or the Mental Health, Alcohol and Other Drugs (MHAOD) service in the Cairns & Hinterland Hospital and Health Service (Cairns & Hinterland HHS) in Queensland. Participants were eligible if they were aged 18 years or older and receiving support for mental health challenges or problematic substance use. Exclusion criteria included risk factors that did not receive medical clearance for participation (e.g., heart condition, injuries etc.), or a diagnosed eating disorder that may be exacerbated by exercise. The AEP contacted referred participants to arrange an initial meeting at the venue to complete written informed consent for participation, medical screening, and baseline assessments. Intake and assessments were conducted throughout the 3-year implementation period (i.e., ‘rolling’ intake).

### Intervention

After induction, participants were offered up to four individual gym sessions with the AEP prior to group-based sessions, depending upon their exercise confidence and complexity of their health conditions. Individual sessions involved exercise technique instruction, education about exercise benefits, and individual programming including aerobic, strength and flexibility exercises tailored to personal health goals, abilities, and sporting interests where applicable. Although participants were primarily engaged for mental health and/or substance use reasons, participants set supplementary goals related to physical health, e.g., ‘getting strong’ or ‘losing fat’. After completion of individual exercise sessions, participants were offered weekly group-based exercise and health education sessions and encouraged to attend at least once per week; participants could attend additional sessions according to their availability and interest.

Group sessions were two hours in duration with up to 10 participants, comprising a one-hour exercise component and a one-hour participatory engagement and health literacy component. The exercise component involved participants completing individually prescribed exercise programs in the gym supervised by the AEP. The AEP monitored participants while providing encouragement and guidance, technique instruction, and advising on training intensity using the 10-point rating of perceived exertion (RPE) scale (moderate intensity is 5–6 out of 10) (Borg, 1998). Aerobic exercises included stationary bike, treadmill, rowing ergometer and elliptical machine; parameters (e.g., speed, resistance) were chosen according to individual participant capacity and preference, and duration ranged between 10 and 30min. Resistance exercises included six to eight exercises completed over 20–40min, targeting main muscle groups such as legs, arms/shoulders, and core, using body weight (e.g., lunges, push-ups) or standard gym equipment (e.g., dumbbells, resistance bands, pin-loaded weight machines). Specific rehabilitation exercises (e.g., to treat shoulder or low back pain) were prescribed as needed for each participant. Stretching was completed in the final 5–10min. Participants were encouraged to work together (‘buddying up’) where goals, personality, and fitness levels aligned. The participatory engagement and health literacy component was co-facilitated by the AEP and dietitian, peer worker, or psychologist provided by partnering organisations, and involved: (i) reflective group discussion, education, and motivational strategies related to improving health and wellbeing; and (ii) practical cooking with the dietitian.

### Data Collection

Participants completed assessments facilitated by the AEP at baseline (T1) and at three follow-up timepoints (T2-T4), each separated by approximately 10–12 weeks (each quarter). Attendance to each group session and the number of days between each assessment point was recorded.

Baseline assessments included self-reported mental health diagnoses, other medical co-morbidities, living situation, employment status, and smoking status. Participants were asked to report any changes in these health and demographic characteristics at each reassessment point. Outcome measures completed at each assessment point are described below.

#### Quality of Life

The Assessment of Quality of Life (AQoL-6D) scale consists of 20 questions assessing six dimensions of mental and physical health: physical health dimensions include independent living, pain, and senses, and mental health dimensions include mental health, coping and relationships. Each dimension has moderate-high reliability (Cronbach’s α = 0.61–0.96), and a demonstrated ability to distinguish general population scores from people with mental illness. The utility score represents combined overall QoL; scores are placed on a scale of 0 to 1, where a score of 1 is indicative of optimal health (Richardson et al., 2011).

#### Social Support

The social support survey consists of questions relating to practical, emotional, and informational support. Practical and emotional support was assessed through family and friend connections by asking about frequency of contact and quality of relationships using questions adapted from a social support questionnaire (Saha et al., 2012). Frequency of contact *(including visits*,* phone calls*,* letters*,* or electronic messages)* was assessed using a 5-point scale (*less than once a month or never*; *1 to 3 days a month*; *1 to 2 days a week*; *3 to 4 days a week*; *most or all days*). Quality of family or friend relationships was assessed by asking how many of those people they could *rely on if you need help* (practical social support) and *confide in if something is troubling you* (emotional social support); possible responses were on a 4-point scale (*none*; *1–2*; *3–4*; *5 or more*). Informational support was assessed by asking about the frequency of contact with professional supports for mental health. To develop an overall social support score for analysis, the frequency of contact was multiplied by quality and summed across practical, emotional and information support domains; scores could range from 0 to 28.

#### Sense of Belonging

The Sense of Belonging Instrument (SOBI) psychological sense subscale (Hagerty et al., 1995) consists of 18 items. Scoring is on a 4-point scale ranging from 1 (Strongly Disagree) to 4 (Strongly Agree), and questions are written in the negative (i.e., high scores represent low sense of belonging). Factor analysis has confirmed that these questions measure related but distinct constructs (*r* = 0.36), and test-retest reliability has been reported for a student cohort (*r* = 0.84) (Hagerty & Patusky, 1995).

#### Physical Activity

The *2-item Physical Activity Questionnaire* (2PAQ) was adapted from the Physical Activity as a Vital Sign (PAVS) questions (Greenwood et al., 2010), which asks about activity at least 30-minutes duration completed in the previous week. The PAVS has been adapted in studies with people with mental illness to focus on an average week (instead of previous week), and of any duration (rather than a minimum duration) (Vancampfort et al., 2016). The 2PAQ was adapted to ask specifically about *purposeful exercise* rather than physical activity: (i) *On average*,* how many days per week do you engage in purposeful exercise (not just incidental)?*; and (ii) *On these days*,* on average*,* how much time do you spend doing this exercise?*. The number of days was multiplied by the duration to estimate weekly exercise.

#### Anthropometric and Physiological Measures

Height was measured to the nearest 0.1 cm using a stadiometer, and weight was measured to the nearest 0.01 kg using electronic scales (Charder MS 6111). Body Mass Index (BMI) was calculated as height divided by weight squared. Blood pressure was measured using an automatic sphygmomanometer.

### Statistical Analyses

To report procedural statistics of service provision (objective 1), number of referrals, participant intake, discontinuation and re-start rates, and number of sessions delivered were summarised for each quarter (3-month period). Discontinuation was defined as not attending any group sessions for at least three months; ‘re-starting’ was defined as attending within the subsequent three months of being defined as discontinued.

To evaluate QoL trajectories (objective 2), linear mixed-effects models were used for total utility score and each dimension (independent living, pain, senses, relationships, mental health, coping) for participants who completed at least two assessment points. Time (representing the duration of participation in years) was included as a fixed effect, and participant IDs were included as random effects (for intercepts and slopes) to account for individual variability in baseline values and rates of change. This approach accounted for repeated QoL measures on participants by creating a regression line for each individual’s QoL trajectory and averaging them to produce a single population-average regression line. To assess potential attrition bias, associations between QoL total utility scores and future missingness were assessed using a repeated-measures analysis of variance (ANOVA), which compared the mean utility score at the first follow-up point (T2) between participants who discontinued after T2, discontinued after T3, or completed T4. A Welch’s *t-*test compared the mean utility score at the second follow-up point (T3) between participants who discontinued after T3 and those who completed T4.

To investigate the association between participant attendance and characteristics on the trajectory of total QoL (objective 3), separate models including each variable of interest (sex, age, BMI, living situation, employment, mental health diagnosis, multimorbidity, social network, sense of belonging, and session density) were examined. These measures were available for retrospective analysis and selected to inform hypothesis development to be explored in future research using more rigorous randomised designs. For interpretability, attendance was converted to a ‘session density’ variable (number of sessions attended/number of days between assessment points * 100) so that every 10% increase corresponded with an additional session every 10 days. Analyses were conducted using R version 4.4.1 (R Core Team, 2023).

## Results

### Objective 1

**Fig. 1:**
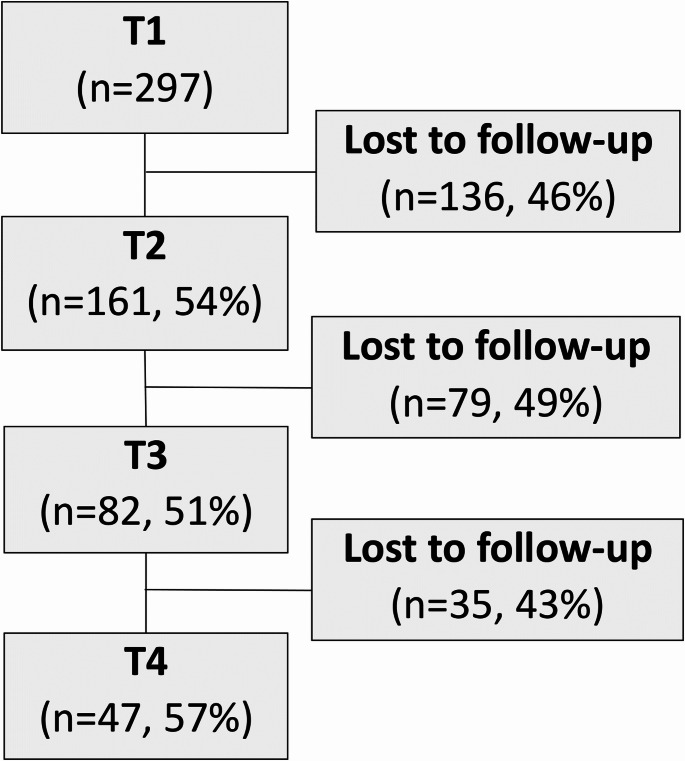
*Left*: Service provision statistics for each quarter (Qtr); *right*: quarterly variation in participant involvement with the service

The service was implemented for three years (12 quarters), and a total of 295 participants were inducted into the service (participant characteristics provided in Supplementary Table 1). The number of active participants each quarter (attending at least once in the ~ 3-month period) ranged from 25 to 61 (mean (M) = 43, standard deviation (SD) = 9). Total attendance for all participants each quarter ranged from 108 to 354 sessions (M = 225, SD = 63), which was an average of 5.2 exercise sessions/quarter/participant (SD = 1.1). Discontinuation rates ranged from 11 to 36 participants/quarter (M = 23, SD = 7), and rates of re-starting with the service ranged from 0 to 8 participants/quarter (M = 3, SD = 2; ~13% of those who discontinued). Quarterly procedural statistics for the service are provided in Fig. 1.

### Objective 2

**Fig. 2:**
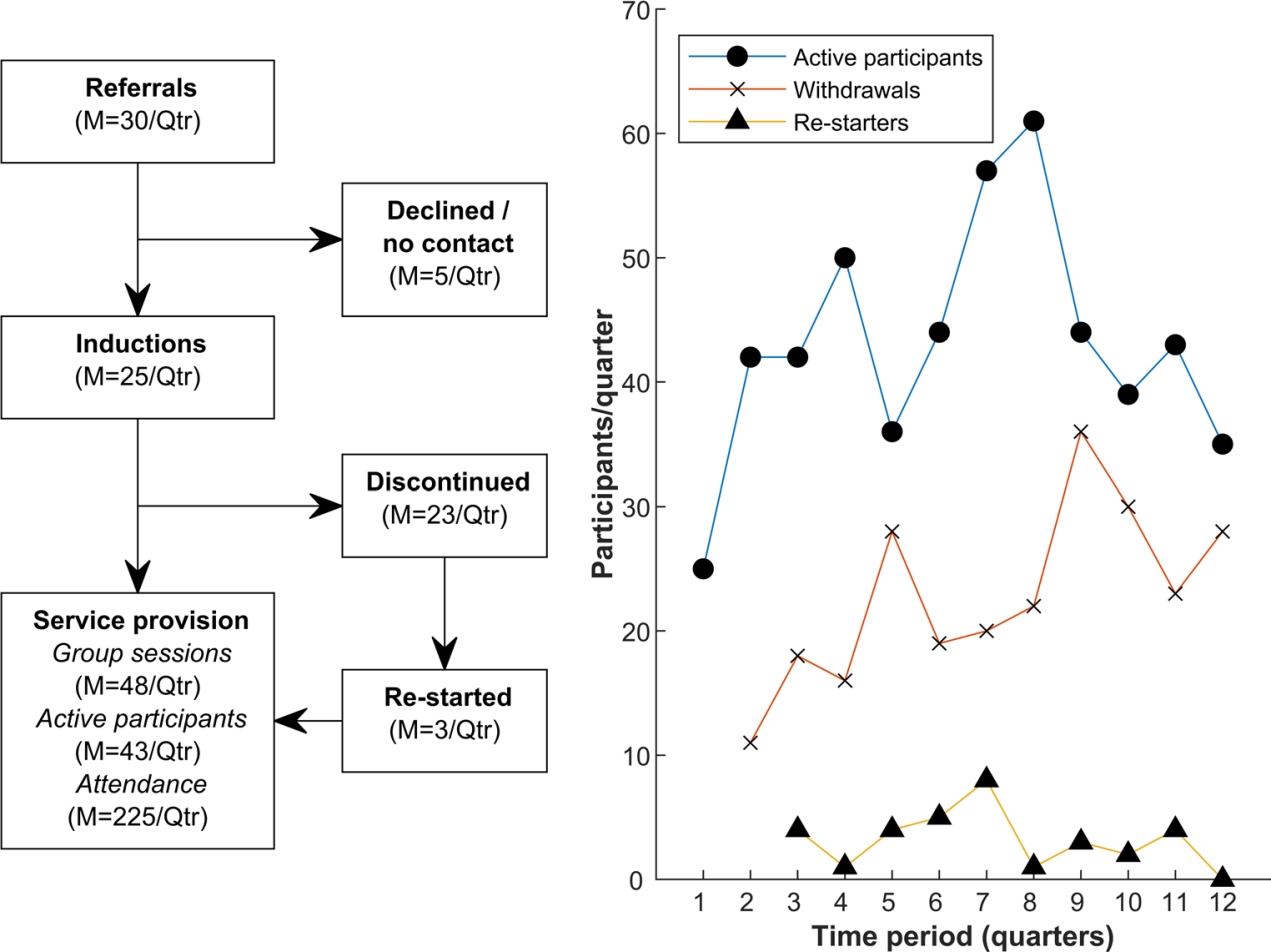
Participant recruitment and retention flow diagram. T1: baseline assessment at induction to the service; T2: first follow-up assessment; T3: second follow-up assessment; T4: third follow-up

Participant retention ranged from 51 to 57% at each of T2, T3 and T4 (Fig. 2). QoL total utility scores at T2 were similar across participants who discontinued after T2, discontinued after T3, or completed T4 (F(82.77) = 0.60; *p* = 0.550). Likewise, QoL total utility scores at T3 were similar for participants who discontinued after T3 or completed T4 (t(75.29) = 0.47; *p* = 0.638), suggesting that QoL was not associated with attrition/retention in the service. Of the 161 participants who completed at least two assessments (T1 and T2), there were slightly more female participants (54.7% female; 43.5% male), with three participants (1.2%) identifying as non-binary. Affective disorder (depression or bipolar disorder) was the most common category of self-reported mental health diagnosis (46.6%) followed by psychotic disorder (32.3%); 15.5% of participants referred through MHAOD service pathways reported no mental health diagnosis. The mean age of participants at baseline was 44 (SD = 13) years, and most participants were non-smokers (*n* = 102, 63.4%). Participant demographics are provided in Table 1.

**Table 1:** Baseline characteristics of participants who completed at least two assessments

	Males (*n* = 70)	Females (*n* = 88)	All (*n* = 161) ^a^
	Mean (SD)	Mean (SD)	Mean (SD)
Age	43.1 (14.2)	44.9 (14.0)	44.0 (14.1)
Body mass index (kg/m^2^)	33.5 (9.2)	31.5 (9.0)	33.0 (9.3)
Self-reported exercise (min/week)	143.9 (239.3)	96.1 (175.5)	121.4 (209.0)
	n (%)	n (%)	n (%)
Meeting physical activity guidelines ^b^	23 (26%)	19 (21%)	44 (27%)
Multimorbidity ^c^	53 (76%)	66 (75%)	121 (75%)
Primary mental health diagnosis ^d^			
*None*	4 (6%)	9 (10%)	25 (16%)
*Psychotic disorder*	36 (51%)	21 (24%)	52 (32%)
-with substance use	3 (8%)	2 (10%)	5 (10%)
*Affective disorder*	23 (33%)	47 (53%)	75 (47%)
-with substance use	3 (13%)	5 (11%)	8 (11%)
*Other* ^d^	7 (10%)	11 (13%)	9 (6%)
-with substance use	1 (14%)	0 (0%)	1 (11%)
Smoking status			
*Non-smoker*	43 (61%)	54 (61%)	102 (63%)
*Smoker*	27 (39%)	34 (39%)	59 (36%)
Living situation			
*Living with significant other/s*	36 (51%)	49 (56%)	84 (52%)
*Living with unrelated people*	14 (20%)	16 (18%)	35 (21%)
*No current place of residence*	1 (1%)	0 (0%)	1 (1%)
*Single person living alone*	19 (27%)	23 (26%)	40 (25%)
Employment status			
*Homemaker/carer*	1 (1%)	10 (11%)	10 (6%)
*No occupation*	51 (73%)	51 (58%)	101 (63%)
*Paid occupation*	11 (16%)	15 (17%)	30 (19%)
*Unpaid occupation*	7 (10%)	11 (13%)	18 (11%)

^a^ Three participants reported non-binary gender; hence, male and female sample sizes do not sum to 161

^b^ Meeting physical activity guidelines defined as self-reporting at least 150 min/week of exercise

^c^ Multimorbidity defined as self-reporting two or more mental or physical health conditions

^d^ Primary diagnosis categorised as ‘psychotic disorder’ if participants self-reported having schizophrenia, schizoaffective disorder or other psychosis; ‘affective disorder’ if self-reported depression or bipolar disorder; and ‘other’ if self-reported personality disorder, post-traumatic stress disorder, anxiety disorder, or eating disorder. Co-occurring alcohol or other substance use disorder is reported as a proportion of participants with the corresponding diagnosis

The mean number of days between assessments was 102.3 (SD = 61.5) days for T1-T2, 138.1 (SD = 101.3) days for T2-T3, and 98.7 (SD = 49.5) for T3-T4. The session density – representing the proportion of days between assessments for which a group session was attended – was M = 8.0% (SD = 5.2%; range = 0–26%) for T1-T2, 5.5% (SD = 5.6%; range = 0–25%) for T2-T3, and 6.9% (SD = 5.8%; range = 0–27%) for T3 to T4. Total attendance for participants who completed at least two assessments was M = 14.7 (SD = 13.0) group sessions; more than half of the sample attended more than 10 group sessions (*n* = 88, 55%), and 35 (22%) attended 20 or more group sessions.

**Fig. 3:**
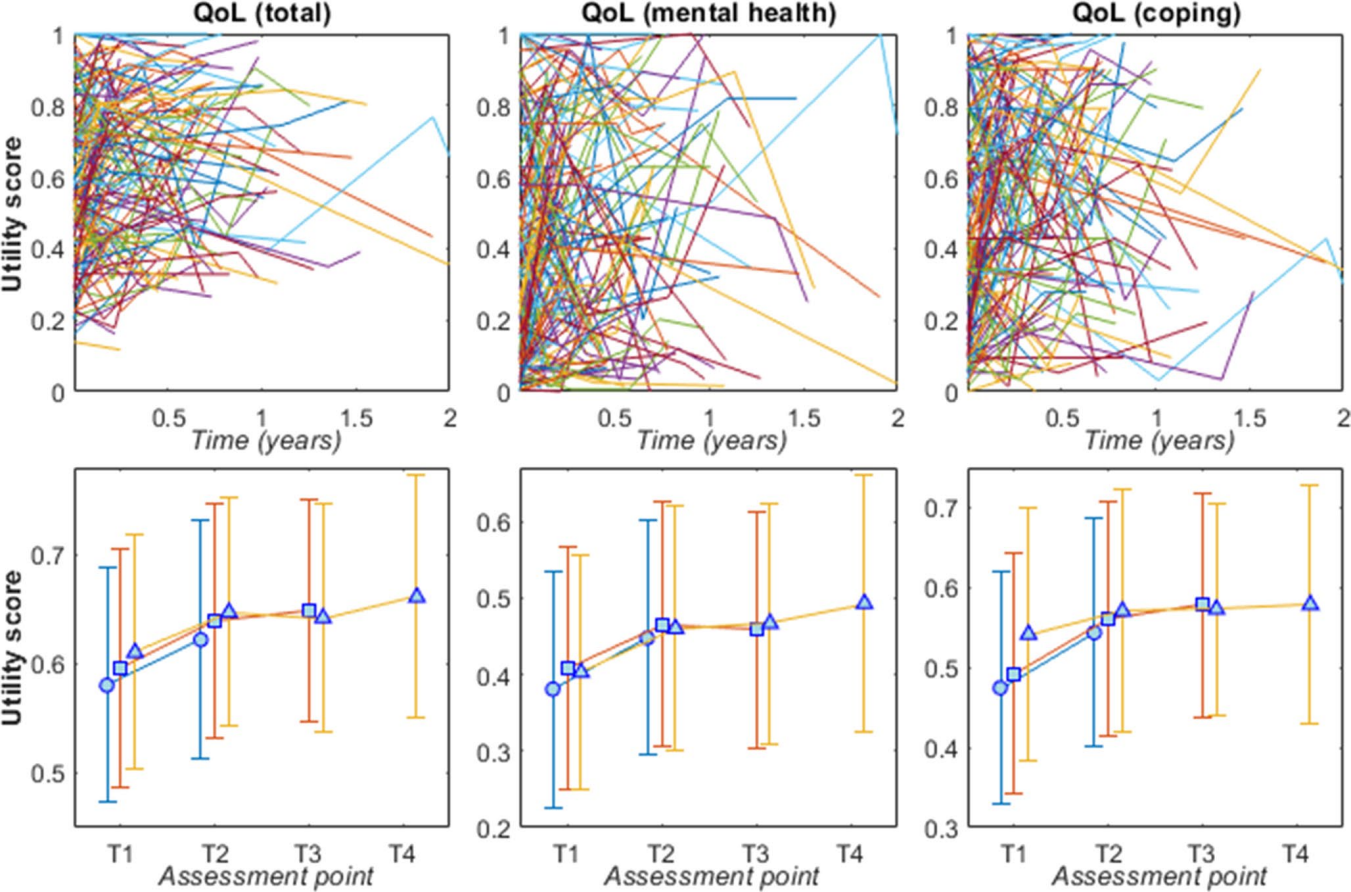
*Upper*: Individual quality of life (QoL) trajectories for each participant; *lower*: aggregated mean (markers) and standard deviation (error bars) at each assessment point for participants who completed T2 (circles; *n* = 161), T3 (squares; *n* = 82), and T4 (triangles; *n* = 47). Utility scores are presented for total QoL (left), mental health dimension (middle) and coping dimension (right)

This participant cohort had a positive overall QoL trajectory for total utility score, as well as mental health and coping dimensions (Table 2). Each year of involvement was associated with a 0.058 improvement in QoL total utility score (95%CI: 0.018 to 0.098; *p* = 0.006), which is 5.8% of the possible range of QoL utility scores. Of the QoL dimensions, mental health and coping were associated with a 0.075 (95%CI: 0.014 to 0.136; *p* = 0.017) and 0.073 (95%CI: 0.012 to 0.135; *p* = 0.022) improvement in their respective utility scores per year. Trajectories for total QoL and mental health and coping dimensions are in Fig. 3; statistics are provided in supplementary tables S2-S4.

**Table 2:** Results for linear mixed effects models estimating the mean change in quality-of-life (QoL) per year during service involvement (*n* = 161)

	QoL change/year (point estimate)	95% CI (lower, upper)	*p*-value
Total utility score	0.058	0.018, 0.098	0.006*
Dimension 1: Independent Living	0.011	−0.041, 0.062	0.686
Dimension 2: Relationships	0.039	−0.025, 0.102	0.234
Dimension 3: Mental Health	0.075	0.014, 0.136	0.017*
Dimension 4: Coping	0.073	0.012, 0.135	0.022*
Dimension 5: Pain	0.008	−0.055, 0.070	0.815
Dimension 6: Senses	0.024	−0.004, 0.052	0.095

Note: Quality of life (QoL: total and for each of the six dimensions) are utility scores investigated in separate linear mixed effects models; statistics refer to the time variable, representing duration of service involvement in years. All models contain time as the fixed effect and participant ID as the random effect. Utility scores range from 0 to 1; therefore a 0.05 improvement represents 5% of the QoL range

### Objective 3

Table 3 shows that baseline sense of belonging and social support were associated with 0.012 (95%CI: −0.014 to −0.010; *p* < 0.001) and 0.013 (95%CI: 0.006 to 0.019; *p* < 0.001) higher total QoL utility scores over the duration of service involvement, respectively. Women had a less favourable QoL trajectory compared with men, with each year of service involvement being associated with 0.067 lower utility scores (95%CI: 0.006 to 0.128; *p* = 0.032); supplementary tables S2-S4 present QoL trajectory statistics for males and females. Multimorbidity and self-reported non-psychotic mental illness were associated with 0.122 (95% CI: 0.055 to 0.189; *p* < 0.001) and 0.238 (95%CI: 0.113 to 0.363, *p* < 0.001) lower QoL scores. Session density (for T1-T2), or baseline BMI, age, employment status, and living status were not significantly associated with QoL trajectory. Statistics for each of the linear mixed effects models are shown in Table 3.

**Table 3:** Linear mixed effects models of quality-of-life trajectories and the influence of participant baseline characteristics (*n* = 161)

Models	QoL change/year (point estimate)	95% CI (lower, upper)	*p*-value
Session density			
*Session density (T1-T2)*	0.633	−0.092, 1.359	0.089
*Time*	0.062	0.021, 0.102	0.003*
Age			
*Age*	−0.000	−0.002, 0.002	0.818
*Time*	0.059	0.019, 0.099	0.005*
Body mass index (BMI)			
*BMI*	−0.003	−0.006, 0.001	0.136
*Time*	0.064	0.019, 0.108	0.006*
Living status			
*Living with significant others (ref)*			
*Living with unrelated people*	−0.010	−0.092, 0.071	0.806
*Living alone*	0.039	−0.032, 0.110	0.285
*Time*	0.062	0.021, 0.102	0.004*
Employment status			
*Homemaker/carer (ref)*			
*Unpaid occupation*	0.090	−0.057, 0.236	0.231
*Paid occupation*	0.072	−0.066, 0.209	0.309
*No occupation*	0.107	−0.014, 0.229	0.085
*Time*	0.058	0.018, 0.098	0.005*
Sex			
*Male (ref)*			
*Female*	−0.067	−0.128, −0.006	0.032*
*Time*	0.058	0.017, 0.098	0.006*
Multimorbidity			
*<* *2 chronic conditions (ref)*			
*Multimorbidity (≥ 2 conditions)*	−0.122	−0.189, −0.055	0.001*
*Time*	0.059	0.019, 0.099	0.005*
Self-reported mental health diagnosis			
*No reported diagnosis (ref)*			
*Psychotic disorder*	−0.082	−0.186, 0.022	0.125
*Affective disorder*	−0.227	−0.329, −0.125	< 0.001*
*Other diagnosis (e.g. personality disorder)*	−0.238	−0.363, −0.113	< 0.001*
*Time*	0.055	0.015, 0.096	0.008*
Sense of belonging			
*Sense of belonging* ^a^	−0.012^a^	−0.014, −0.010	< 0.001*
*Time*	0.055	0.015, 0.095	0.008*
Social support			
*Social support*	0.013	0.006, 0.019	<0.001*
Time	0.058	0.018, 0.098	0.006*

**Note: **All models use quality of life (QoL) total utility score as the dependent variable, with time (duration of involvement with the service in years) as the fixed effect, and participant ID as the random effect. ^a^ Sense of belonging questions are negatively worded; therefore, more negative scores represent greater sense of belonging.

## Discussion

The aim of this study was to evaluate changes in QoL while engaged with an AEP-led exercise and healthy lifestyle service for people experiencing mental health challenges or problematic substance use. A positive QoL trajectory was observed over the duration of involvement with the service, which appeared to be driven by improvements in the mental health and coping dimensions. QoL trajectory was associated with higher social support and sense of belonging at baseline, and negatively associated with being female, having self-reported at least two chronic conditions (multimorbidity), or a non-psychotic mental illness. While changes are unable to be attributed to the intervention in this retrospective evaluation, these findings can inform further research into the mediators and moderators of QoL, as well as the design of AEP services to better accommodate the needs of this group.

Total QoL improved by 0.058 per year (5.8% of the scoring range), with the greatest improvements being in the coping and mental health dimensions (0.073 and 0.075, respectively). These trends were significant despite the wide range of QoL scores leading to large variance in the outcome, possibly caused by the broad inclusion criteria and diverse sample. Improvements in QoL are consistent with previous reviews have demonstrated that exercise interventions can improve QoL (measured using a range of QoL measures) in people with a range of mental health challenges or problematic substance use (Solmi et al., 2025; Vancampfort et al., 2025). One implementation study that used the AQoL-6D measure to evaluate a 12-week AEP led intervention for a youth mental health service also reported improvements in total QoL scores (*d* = 0.61), as well as independent living (d = 0.8), mental health (d = 0.67) and coping (d = 0.76) dimensions (Lederman et al., 2020). Independent living did not significantly improve in our study, likely because participants were community-dwelling adults. Exercise can be used as a positive coping strategy for distress (Holubova et al., 2015), as well as a meaningful activity to assist with recovery (Soundy et al., 2011), making community-based exercise a valuable adjunctive treatment for supporting people with mental health challenges and problematic substance use. Community-based, AEP led interventions may facilitate improvements in mental health, coping and overall quality of life, which could foreseeably reduce relapse and hospital readmission in this group.

Social support and sense of belonging at baseline were associated with a positive trajectory of total QoL, highlighting the relationship between social connectedness and health outcomes (Santini et al., 2015; Uchino, 2006). While this association was relatively small (0.012–0.013), it suggests that higher social support and sense of belonging are important influences for improvement in quality of life and recovery in people with mental health challenges and problematic substance use. This population commonly experiences social isolation and loneliness, contributing to psychological distress and the symptoms of mental illness and addiction (Richter et al., 2019). Social network size (number of connections) and quality (satisfaction) has previously been shown to be associated with better mental QoL in people with severe mental illness (Hansson, 2006) and substance use (Armoon et al., 2022). Group-based community activities can increase social interaction and subsequently improve social support for people with mental illness, and exercise itself has been shown to increase willingness to socially interact with others and improve positive perceptions of socialisation in individuals with mental illness (Brand et al., 2018). Group exercise sessions with professional supervision which are delivered in a welcoming environment may enhance social support and sense of belonging in this group.

Session density between T1-T2 was not significantly associated with QoL trajectory. This suggests that a larger sample size or greater ‘dose’ of attendance may be needed to demonstrate a relationship with QoL changes. While approximately 3% (*n* = 5) participants had session density of over 20% at each timepoint (representing one session every 5 days), the average session density was 5–10% between assessments (one every 10–20 days). Future studies on interventions using a higher frequency of sessions, and using a randomised controlled trial design, should investigate the contribution of session density to QoL outcomes. Goal setting in this program was informal, involving identification of reasons for participating and tailoring exercise programs accordingly; however, strategies to improve attendance could include evidence-based goal setting including preparation, goal setting, planning and follow-up phases (Bird et al., 2024). Participants who had support workers accompanying them to the sessions (e.g., case managers, non-clinical staff) or highly engaged family, were observed to attend more consistently. Some support workers actively participated in the education sessions or provided encouragement during gym sessions, resulting in better adoption of advice from the AEP, whereas others were less actively engaged in supporting their clients. Training for mental health staff in mindfulness and engagement strategies may assist in supporting their clients with mental health challenges and problematic substance use to actively participate in community activities.

Participant characteristics had notable associations with QoL trajectories. Having multimorbidity, non-psychotic mental illness, and being female were all associated with less favourable QoL trajectories in the participant cohort. Compared with males, more female participants had non-psychotic mental illnesses (66% vs. 51%), and a higher proportion of females identified as a homemaker/carer (11% vs. 1%), which may suggest potential health and sociodemographic interactions. While this hypothesis-generating research is unable to ascertain the influences on QoL trajectories for these groups, it was observed that social determinants significantly impacted the level of engagement for some participants. For example, contacting some participants was challenging because of housing instability, inadequate access to affordable transport, or limited access to mobile phones and internet. Further, some participants lacked appropriate attire for the gym (e.g., shoes, active wear), which was a barrier to attendance. These barriers were able to be effectively overcome by proactive support workers who made regular contact with the AEP and addressed the needs of their clients where possible. Further research into addressing social disadvantage of people with mental health challenges and problematic substance use, and the corresponding impact on engagement with health-enhancing interventions, should be conducted.

A strength of this study was its high ecological validity. The service was implemented in a regional community and funded by a Primary Health Network, which is a commissioning body of government funding for community health services. The service was available as a fully subsidised healthcare service (i.e., free) to participants, and accessible through established referral pathways between other mental health organisations and services. Almost 300 participants with a range of mental health challenges and problematic substance use entered the service over three years, highlighting the demand for exercise services in regional communities that commonly experience higher rates of preventable chronic disease and lower access to preventative health services. Attrition was approximately 50% for follow-up assessments which were spaced roughly 3-months apart. This is consistent with attrition/retention rates in other mental health and substance use treatment settings: analysis of community alcohol and other drug treatment services in Australia indicated 42.7% retention after 14 weeks (Porter, 2013)^p.80^ and mental healthcare attrition rates in generalist settings range between 43% and 57% internationally (Fernandez et al., 2021). This may reflect a variety of challenges with engagement, including life circumstances, program acceptability, and other barriers reported by the general population (Alyafei and Albaker 2020; Freene et al., 2014) as well as people with mental health challenges and substance use disorders (Firth et al., 2016).

This study was a retrospective analysis of a community exercise service. The absence of a control group limits conclusions about causality. Analyses were therefore hypothesis-generating rather than hypothesis-testing, aiming to characterise changes in QoL during participant engagement with the service. Each potentially influential variable was explored independently using multilevel modelling to inform future research rather than attempting a predictive model. The risk of false positives is higher because of the large numbers of variables being considered. Other limitations include the use of self-report to assess mental health diagnoses which is subject to recall error, and the lack of assessment of other clinical variables, such as trauma, or alcohol or substance use. Only primary mental health diagnosis was included in linear mixed models which was determined by categorising self-reported diagnoses into disorder groups that tend to be most severe and enduring, i.e., if depression and schizophrenia were reported, participants were categorised as having a primary psychotic disorder. The influence of diagnosis on QoL trajectory should therefore be interpreted with caution. For this retrospective evaluation, social support was quantitatively assessed using an unvalidated questionnaire. More rigorous hypothesis-testing research should be conducted to determine the repeatability of findings using validated instruments and clinically confirmed diagnosis, and evaluating implementation factors outlined in global recommendations (Teasdale, 2025) to advance the field.

## Conclusion

QoL trajectories for people with a range of mental health challenges and problematic substance use improved after beginning an exercise and healthy lifestyle program delivered by an AEP. Social support and sense of belonging were associated with positive changes, and the increase in total QoL appeared to be driven by improvements in coping and mental health dimensions. Holistic community-based support may improve health outcomes in community-dwelling individuals and potentially prevent hospitalisations in the long term. Some characteristics were associated with less favourable trajectories, such as having multimorbidity, being female, or reporting a non-psychotic mental illness, highlighting the need to target interventions to address the specific needs of these groups. While attendance was comparable with general mental healthcare, incorporating additional social and professional support strategies to overcome barriers to attendance, evidence-based goal setting, and offering more frequent sessions may further improve outcomes. Further implementation research using randomised designs is needed on AEP-led interventions as an essential support pathway for people with mental health challenges and problematic substance use.

## Supplementary Information

Below is the link to the electronic supplementary material.

Supplementary Material 1 (DOCX. 50.4 KB)

## Data Availability

No datasets were generated or analysed during the current study.

## References

[CR1] Alyafei, A., & Albaker, W. (2020). Barriers and facilitators for regular physical exercise among adult females narrative review 2020. *Journal of Community Medicine and Public Health,**4*(178), 2577–2228100078.

[CR2] Armoon, B., Fleury, M. J., Bayat, A. H., Bayani, A., Mohammadi, R., & Griffiths, M. D. (2022). Quality of life and its correlated factors among patients with substance use disorders: A systematic review and meta-analysis. *Archives of Public Health*, *80*(1), 179. 35927697 10.1186/s13690-022-00940-0 PMC9351239

[CR3] Bird, M. D., Swann, C., & Jackman, P. C. (2024). The what, why, and how of goal setting: A review of the goal-setting process in applied sport psychology practice. *Journal of Applied Sport Psychology*, *36*(1), 75–97.

[CR4] Borg, G. (1998). *Borg’s perceived exertion and pain scales*. Human kinetics.

[CR5] Brand, S., Colledge, F., Ludyga, S., Emmenegger, R., Kalak, N., Bahmani, S., Holsboer-Trachsler, D., Pühse, E., U., & Gerber, M. (2018). Acute bouts of exercising improved mood, rumination and social interaction in inpatients with mental disorders. *Frontiers in Psychology*, *9*, 249. 29593592 10.3389/fpsyg.2018.00249 PMC5859016

[CR6] Chapman, J. J., Fraser, S. J., Brown, W. J., & Burton, N. W. (2016). Physical activity preferences, motivators, barriers and attitudes of adults with mental illness. *Journal of Mental Health*, *25*(5), 448–454. 27049695 10.3109/09638237.2016.1167847

[CR7] Connell, J., Brazier, J., O’Cathain, A., Lloyd-Jones, M., & Paisley, S. (2012). Quality of life of people with mental health problems: A synthesis of qualitative research. *Health and Quality of Life Outcomes*, *10*, 1–16. 23173689 10.1186/1477-7525-10-138 PMC3563466

[CR8] R Core Team (2023). *R: A Language and Environment for Statistical Computing*. In (Version R version 4.2.3) R Foundation for Statistical Computing. https://www.R-project.org/

[CR9] Evans, S., Banerjee, S., Leese, M., & Huxley, P. (2007). The impact of mental illness on quality of life: A comparison of severe mental illness, common mental disorder and healthy population samples. *Quality of Life Research*, *16*, 17–29. 17036252 10.1007/s11136-006-9002-6

[CR10] Exercise & Sports Science Australia (2024). *Accredited Exercise Physiologist Scope of Practice*. https://www.essa.org.au/Common/Uploaded%20files/Standards/AEP%20Scope%20of%20Practice.pdf

[CR11] Fernandez, D., Vigo, D., Sampson, N. A., Hwang, I., Aguilar-Gaxiola, S., Al-Hamzawi, A. O., Alonso, J., Andrade, L. H., Bromet, E. J., & de Girolamo, G. (2021). Patterns of care and dropout rates from outpatient mental healthcare in low-, middle-and high-income countries from the World Health Organization’s world mental health survey initiative. *Psychological Medicine,**51*(12), 2104–2116. 32343221 10.1017/S0033291720000884 PMC8265313

[CR12] Fibbins, H., Edwards, L., Morell, R., Lederman, O., Ward, P., & Curtis, J. (2021). Implementing an exercise physiology clinic for consumers within a community mental health service: A real-world evaluation. *Frontiers in Psychiatry,**12*, 791125. 34899443 10.3389/fpsyt.2021.791125 PMC8651871

[CR13] Firth, J., Rosenbaum, S., Stubbs, B., Gorczynski, P., Yung, A., & Vancampfort, D. (2016). Motivating factors and barriers towards exercise in severe mental illness: A systematic review and meta-analysis. *Psychological Medicine,**46*(14), 2869–2881. 10.1017/S0033291716001732 27502153 10.1017/S0033291716001732 PMC5080671

[CR14] Freene, N., Waddington, G., Chesworth, W., Davey, R., & Cochrane, T. (2014). Community group exercise versus physiotherapist-led home-based physical activity program: Barriers, enablers and preferences in middle-aged adults. *Physiotherapy Theory and Practice*, *30*(2), 85–93. 24405399 10.3109/09593985.2013.816894

[CR15] Furzer, B. J., Wright, K. E., Edoo, A., & Maiorana, A. (2021). Move your mind: Embedding accredited exercise physiology services within a hospital-based mental health service. *Australasian Psychiatry*, *29*(1), 52–56. 32722965 10.1177/1039856220943030

[CR16] Greenwood, J. L. J., Joy, E. A., & Stanford, J. B. (2010). The physical activity vital sign: A primary care tool to guide counseling for obesity. *Journal of Physical Activity & Health,**7*(5), 571–576. 10.1123/jpah.7.5.571 20864751 10.1123/jpah.7.5.571

[CR17] Hagerty, B. M., & Patusky, K. (1995). Developing a measure of sense of belonging. *Nursing Research,**44*(1), 9–13. 7862549

[CR18] Hansson, L. (2006). Determinants of quality of life in people with severe mental illness. *Acta Psychiatrica Scandinavica*, *113*, 46–50. 10.1111/j.1600-0447.2005.00717.x 16445482

[CR19] Holubova, M., Prasko, J., Hruby, R., Kamaradova, D., Ociskova, M., Latalova, K., & Grambal, A. (2015). Coping strategies and quality of life in schizophrenia: Cross-sectional study. *Neuropsychiatric Disease And Treatment,**11*(null), 3041–3048. 10.2147/NDT.S96559 26677331 10.2147/NDT.S96559 PMC4677764

[CR20] Korman, N., Fox, H., Skinner, T., Dodd, C., Suetani, S., Chapman, J., Parker, S., Dark, F., Collins, C., & Rosenbaum, S. (2020). Feasibility and acceptability of a student-led lifestyle (diet and exercise) intervention within a residential rehabilitation setting for people with severe mental illness, GO HEART (Group occupation, health, exercise and rehabilitation treatment). *Frontiers in Psychiatry,**11*, 319. 10.3389/fpsyt.2020.00319 32411024 10.3389/fpsyt.2020.00319 PMC7198865

[CR21] Korman, N., Stanton, R., Vecchio, A., Chapman, J., Parker, S., Martland, R., Siskind, D., & Firth, J. (2023). The effect of exercise on global, social, daily living and occupational functioning in people living with schizophrenia: A systematic review and meta-analysis. *Schizophrenia Research*, *256*, 98–111. 37209456 10.1016/j.schres.2023.04.012

[CR22] Lederman, O., Ward, P. B., Rosenbaum, S., Maloney, C., Watkins, A., Teasdale, S., Morell, R., & Curtis, J. (2020). Stepping up early treatment for help-seeking youth with at-risk mental states: Feasibility and acceptability of a real-world exercise program. *Early Intervention in Psychiatry,**14*(4), 450–462. 10.1111/eip.12871 31531959 10.1111/eip.12871

[CR23] Lund, K., Argentzell, E., Leufstadius, C., Tjörnstrand, C., & Eklund, M. (2019). Joining, belonging, and re-valuing: A process of meaning-making through group participation in a mental health lifestyle intervention. *Scandinavian Journal of Occupational Therapy,**26*(1), 55–68. 29179630 10.1080/11038128.2017.1409266

[CR24] Porter, M. R. (2013). An analysis of treatment retention and attrition in an Australian therapeutic community for substance abuse treatment.

[CR25] Richardson, J., Sinha, K., Iezzi, A., & Khan, M. (2011). Modelling the utility of health states with the Assessment of Quality of Life (AQoL) 8D instrument: overview and utility scoring algorithm. *Research Paper 63, Center for Health Economics*.

[CR26] Richter, D., & Hoffmann, H. (2019). Social exclusion of people with severe mental illness in Switzerland: Results from the Swiss health survey. *Epidemiology and Psychiatric Sciences,**28*(4), 427–435. 29233203 10.1017/S2045796017000786 PMC6998964

[CR27] Saha, S., Scott, J., Varghese, D., & McGrath, J. (2012). Social support and delusional-like experiences: A nationwide population-based study. *Epidemiology and Psychiatric Sciences*, *21*(2), 203–212. 22789170 10.1017/S2045796011000862

[CR28] Santini, Z. I., Koyanagi, A., Tyrovolas, S., Mason, C., & Haro, J. M. (2015). The association between social relationships and depression: A systematic review. *Journal Of Affective Disorders,**175*, 53–65. 10.1016/j.jad.2014.12.049 25594512 10.1016/j.jad.2014.12.049

[CR29] Seymour, J., Pratt, G., Patterson, S., Korman, N., Rebar, A., Tillston, S., & Chapman, J. (2021). Changes in self-determined motivation for exercise in people with mental illness participating in a community-based exercise service in Australia. *Health and Social Care in the Community*. 10.1101/2019.12.11.12345678 34614232 10.1111/hsc.13588

[CR30] Solmi, M., Basadonne, I., Bodini, L., Rosenbaum, S., Schuch, F. B., Smith, L., Stubbs, B., Firth, J., Vancampfort, D., & Ashdown-Franks, G. (2025). Exercise as a transdiagnostic intervention for improving mental health: An umbrella review. *Journal of Psychiatric Research* 184:91–101. 10.1016/j.jpsychires.2025.02.024 40043589 10.1016/j.jpsychires.2025.02.024

[CR31] Soundy, A., Kingstone, T., & Coffee, P. (2011). Understanding the psychosocial processes of physical activity for individuals with severe mental illness: A meta-ethnography. In *Mental Illnesses - Evaluation, Treatments and Implications* (pp. 3–20). InTech. 10.5772/2476

[CR32] Teasdale, S., Curtis, J., Ward, P., Watkins, A., Rosenbaum, S., Morell, R., & Fibbins, H. (2024). *Keeping the body in mind: The evolution of a lifestyle program for mental health consumers*. Mindgardens Neuroscience Network.

[CR33] Teasdale, S., Machaczek, K., Marx, W., Eaton, M., Chapman, J., Milton, A., Oyeyemi, A., Pelupessy, D., Schuch, F., Gatera, G., Ahmed, H., Diatri, H., Jidda, I., Gutiérrez-Peláez, M., Elshazly, M., Fugu, M., Grinko, N., Indu, P., Oo, S., … and the Lancet Psychiatry Physical Health Commission Consortium. (2025). Implementing lifestyle interventions in mental health care: Third report of the Lancet Psychiatry Physical Health Commission. *The Lancet Psychiatry*. 10.1016/S2215-0366(25)00170-1 40812962 10.1016/S2215-0366(25)00170-1

[CR34] Tweed, L. M., Rogers, E. N., & Kinnafick, F. E. (2021). Literature on peer-based community physical activity programmes for mental health service users: A scoping review. *Health Psychology Review*, *15*(2), 287–313. 10.1080/17437199.2020.1715812 31937185 10.1080/17437199.2020.1715812

[CR35] Uchino, B. N. (2006). Social support and health: A review of physiological processes potentially underlying links to disease outcomes. *Journal of Behavioral Medicine*, *29*(4), 377–387. 10.1007/s10865-006-9056-5 16758315 10.1007/s10865-006-9056-5

[CR36] Vancampfort, D., Rosenbaum, S., Schuch, F. B., Ward, P. B., Probst, M., & Stubbs, B. (2016a). Prevalence and predictors of treatment dropout from physical activity interventions in schizophrenia: A meta-analysis. *General Hospital Psychiatry*, *39*, 15–23. 10.1016/j.genhosppsych.2015.11.008 26719106 10.1016/j.genhosppsych.2015.11.008

[CR37] Vancampfort, D., Stubbs, B., Probst, M., De Hert, M., Schuch, F. B., Mugisha, J., Ward, P. B., & Rosenbaum, S. (2016b). Physical activity as a vital sign in patients with schizophrenia: Evidence and clinical recommendations. *Schizophrenia Research,**170*(2), 336–340. 10.1016/j.schres.2016.01.001 26782636 10.1016/j.schres.2016.01.001

[CR38] Vancampfort, D., Firth, J., Stubbs, B., Schuch, F., Rosenbaum, S., Hallgren, M., Deenik, J., Ward, P. B., Mugisha, J., & Van Damme, T. (2025). The efficacy, mechanisms and implementation of physical activity as an adjunctive treatment in mental disorders: A meta-review of outcomes, neurobiology and key determinants. *World Psychiatry,**24*(2), 227–239. 40371806 10.1002/wps.21314 PMC12079350

[CR39] Whiteford, H. A., Degenhardt, L., Rehm, J., Baxter, A. J., Ferrari, A. J., Erskine, H. E., Charlson, F. J., Norman, R. E., Flaxman, A. D., & Johns, N. (2013). Global burden of disease attributable to mental and substance use disorders: Findings from the Global Burden of Disease Study 2010. *Lancet,**382*(9904), 1575–1586. 23993280 10.1016/S0140-6736(13)61611-6

[CR40] Whybird, G., Nott, Z., Savage, E., Korman, N., Suetani, S., Hielscher, E., Vilic, G., Tillston, S., Patterson, S., & Chapman, J. (2022). Promoting quality of life and recovery in adults with mental health issues using exercise and nutrition intervention. *International Journal of Mental Health*, *51*(4), 424–447.

