# Correction to: Quality of Life of People with Mental Health Challenges and Problematic Substance Use while Engaged with an Exercise Physiology Service

**DOI:** 10.1007/s10597-025-01528-y

**Published:** 2025-10-10

**Authors:** Jane Kugelman, Meg Doohan, Brett Dyer, Jake O’Brien, Mridula Kayal, Justin Chapman

**Affiliations:** 1 School of Public Health, University of Queensland, Brisbane, Australia; 2 Centre for Mental Health, School of Pharmacy and Medical Sciences, Griffith University, Brisbane, Australia; 3 Griffith Biostatistics Unit, Griffith University, Brisbane, Australia; 4 Department of Gastroenterology and Hepatology, Metro South Health, Brisbane, Australia; 5 Mental Health Alcohol Tobacco and Other Drugs Service, Cairns and Hinterland Hospital and Health Service, Brisbane, Australia; 6 Addiction and Mental Health Service, Metro South Health, Brisbane, Australia

**Correction to: Community Mental Health Journal**

10.1007/s10597-025-01518-0

In the original version of this article, the given and family names of second author were incorrectly structured.

The author name should have been Meg Doohan instead of Doohan Meg.

The original article has been corrected.

