# Supplementary material for: Quality of Life of People with Mental Health Challenges and Problematic Substance Use while Engaged with an Exercise Physiology Service
Source: Community Ment Health J. 2025 Sep 18;62(2):245–56. doi: 10.1007/s10597-025-01518-0 (PMC12852210; doi:10.1007/s10597-025-01518-0)
Supplement: Supplementary file 1 — Supplementary Material 1 (DOCX. 50.4 KB) [file 10597_2025_1518_MOESM1_ESM.docx]

| **SUPPLEMENTARY INFORMATION**  **Appendix A**   \| The aim was always to improve access to exercise support for community dwelling adults (over 18 years) with a range of mental health issues, in partnership with mental health services and organisations. The vision was to embed an integrated service model for provision of physical activity and exercise support into routine mental healthcare.  In this Australian example, a peer worker developed and implemented exercise and peer support programs with public mental health services and non-government organisations (NGO). This program expanded under a service innovation grant, before adapting to administration under federal disability funding, then becoming a service model commissioned by a Primary Health Network (PHN).  Some parameters remained the same throughout this evolution: it has always been face-to-face, involving individualised intake, assessment and programming with an exercise professional, and gym-based exercise instruction in groups of up to 10 participants for 1-2 sessions/week.  The program has always been delivered at community gym facilities of a not-for-profit sports and recreation organisation, with sessions held during off-peak times (10am-4pm). Sessions are 2-hours in duration, involving group-based exercise in the gym for 60-min, and social support, health and nutritional education and group discussion in a meeting space for 60-min.  Some program components have changed depending on funding source: (i) the program initially involved peer support and personal training, (ii) with an innovation grant this expanded to include cooking and nutritional education using chefs, (iii) accredited exercise physiologists (AEPs) and dietitians became the primary program facilitators under federal disability funding, (iv) finally, AEPs, dietitians, and peer support workers co-facilitate the program under PHN commissioning and in-kind support from organisational partners.  Funding stability impacted the program structure. When funding was short-term (e.g. 12-month contracts) or fee-for-service based (federal disability funding), the program was 8-weeks in duration with sessions once/week. When the program was commissioned in a block funding arrangement, participants could elect to do 1-2 sessions/week on an ongoing basis with re-assessments completed by the AEP every 8-10-weeks.  The program materials were updated to accommodate these changes to program structure. (1) Under an 8-week program structure, the exercise professional focused on upskilling participants to improve knowledge and confidence for gym-based exercise. Participants followed a structured program designed to give them exposure to a range of exercises and training types with individualisation provided by the exercise professional, and participants were given a gym membership to support independent exercise outside the supervised sessions. (2) Under the ongoing service arrangement, participants had up to four individual sessions with the AEP to develop their individualised program and improve gym knowledge and confidence, then they completed their individualised program in a group exercise environment. Gym memberships weren’t provided because participants could attend on an ongoing basis. The AEP updated their program as needed and re-assessed them every 8-10 weeks.  Funding administration also impacted the organisational relationships. NGOs and the public mental health service partnered with the not-for-profit sports and recreation organisation for the innovation grant, and the program acted as a referral pathway. To access federal disability funding, a NGO co-facilitated the program with the not-for-profit sports and recreation organisation: the not-for-profit sports and recreation organisation provided the facility and AEP, and the NGO provided the dietitian and administered the program funding. Administering the program under federal disability funding proved to be unsustainable; however, evidence generated from these programs supported a successful tender application. Under service commissioning arrangements, partnership with the NGOs and public mental health service were formalised using Collaborative Deeds and Memorandum of Understanding agreements. Seeing the value of the program for consumers, organisational partners contributed staff time in-kind to cofacilitate the program and strengthen referral pathways. \| \| --- \| \| Barriers primarily related to funding availability and stability, and enablers primarily related to the good-will and commitment of front-line and management staff in the mental health and not-for-profit sectors. The program was developed in 2012 by a peer worker, who continued to adapt and manage the program under concurrent employed positions with three different organisations until 2023. Successful elements have been (1) evaluation, (2) partnership, and (3) leadership.  Qualitative and quantitative evaluation demonstrated feasibility and impact, supporting subsequent funding applications. Partnership for co-facilitation of the program strengthened referral pathways, led to creative solutions for accessing different funding schemes, and increased participant engagement through multidisciplinary involvement. Leadership in the form of championing the program to partners and senior management was integral to forming cross-sectoral partnerships and implementing robust evaluation plans, and for continuing the momentum of each project phase into subsequent funding and program arrangements.  The program/service has reached over 600 participants statewide in Queensland, Australia. Improvements in quality of life, recovery, motivation for exercise and sense of belonging have been evidence in the evaluation. Participants also reported that key elements related to the success of the program have been: (i) the support and welcoming environment afforded by the partnering community organisations; (ii) group-based program with people from similar backgrounds and experiences; (iii) personable, approachable, and compassionate program facilitators. Participants reported a range of benefits to their mental and physical health, including unexpected benefits such as improved relationships, self-worth, and confidence in pursuing new opportunities (e.g. employment). \|   **Table S1** Baseline characteristics of participant groups who completed each assessment timepoint (T1-T4) | | | | |
| --- | --- | --- | --- | --- | --- | --- |
| Characteristic | Sample with T1 | Sample with T1 & T2 | Sample with T1-T3 | Sample with T1-T4 |
|  | (n=295) | (n=161) | (n=82) | (n=47) |
|  | *M (SD)* | *M (SD)* | *M (SD)* | *M (SD)* |
| *Age* | 42.1 (13.9) | 44 (14.1) | 45 (15.7) | 46.3 (16.4) |
| *Body mass index (kg/m^2^)* | 32.1 (8.6) | 32.3 (9) | 32.5 (8.6) | 33.8 (8.6) |
|  | *n* (%) | *n* (%) | *n* (%) | *n* (%) |
| *Meeting physical activity guidelines ^b^* | 82 (28%) | 44 (27%) | 22 (27%) | 11 (23%) |
| *Multimorbidity ^c^* | 205 (70%) | 121 (75%) | 62 (76%) | 37 (79%) |
| *Primary mental health diagnosis ^d^* |  |  |  |  |
| None | 30 (10%) | 14 (9%) | 5 (6%) | 3 (6%) |
| Psychotic disorder | 107 (37%) | 58 (36%) | 36 (44%) | 18 (38%) |
| *-with substance use* | 8 (7%) | 5 (9%) | 2 (6%) | 0 (0%) |
| Affective disorder | 115 (40%) | 71 (44%) | 39 (48%) | 25 (53%) |
| *-with substance use* | 16 (14%) | 8 (11%) | 5 (13%) | 3 (12%) |
| Other *^d^* | 39 (13%) | 18 (11%) | 2 (2%) | 1 (2%) |
| *-with substance use* | 6 (15%) | 1 (6%) | 0 (0%) | 0 (0%) |
| *Smoking status* |  |  |  |  |
| Non-smoker | 160 (55%) | 100 (62%) | 49 (60%) | 33 (70%) |
| Smoker | 128 (44%) | 61 (38%) | 33 (40%) | 14 (30%) |
| *Living situation* |  |  |  |  |
| Living with significant other/s | 153 (53%) | 85 (53%) | 39 (48%) | 23 (49%) |
| Living with unrelated people | 47 (16%) | 30 (19%) | 17 (21%) | 8 (17%) |
| No current place of residence | 3 (1%) | 1 (1%) | 1 (1%) | 1 (2%) |
| Single person living alone | 84 (29%) | 44 (27%) | 25 (30%) | 15 (32%) |
| *Employment status* |  |  |  |  |
| Homemaker/carer | 18 (6%) | 11 (7%) | 5 (6%) | 3 (6%) |
| No occupation | 187 (64%) | 104 (65%) | 58 (71%) | 30 (64%) |
| Paid occupation | 54 (19%) | 26 (16%) | 11 (13%) | 7 (15%) |
| Unpaid occupation | 25 (9%) | 18 (11%) | 8 (10%) | 7 (15%) |

**Tables S2-S4** provide outcome data for each participant group who had T1 & T2 (**Table S2**), T1-T3 (**Table S3**), and T1-T4 (**Table S4**), split by male and female participants

**Note**: three participants reported non-binary gender; hence, female and male samples do not sum to the total sample. Any further difference is due to missing data.

| **Table S2: Outcome data for participants with at least T1-T2 data** | | | | | |
| --- | --- | --- | --- | --- | --- |
|  |  | **T1** | **T2** | **T3** | **T4** |
| ***Sample sizes*** |  | ***n*** | ***n*** | ***n*** | ***n*** |
|  | All | 161 | 161 | - | - |
|  | Female | 88 | 88 | - | - |
|  | Male | 70 | 70 | - | - |
| ***Outcomes*** |  | ***Mean (SD)*** | ***Mean (SD)*** | ***Mean (SD)*** | ***Mean (SD)*** |
| *Quality of Life* | |  |  |  |  |
| Total utility score | All | 0.58 (0.21) | 0.62 (0.22) | - | - |
|  | Female | 0.55 (0.19) | 0.59 (0.21) | - | - |
|  | Male | 0.62 (0.24) | 0.66 (0.23) | - | - |
| Independent living | All | 0.73 (0.27) | 0.75 (0.26) | - | - |
|  | Female | 0.74 (0.25) | 0.75 (0.25) | - | - |
|  | Male | 0.73 (0.27) | 0.75 (0.26) | - | - |
| Relationships | All | 0.6 (0.3) | 0.61 (0.3) | - | - |
|  | Female | 0.59 (0.29) | 0.62 (0.3) | - | - |
|  | Male | 0.6 (0.31) | 0.6 (0.3) | - | - |
| Mental Health | All | 0.38 (0.31) | 0.45 (0.31) | - | - |
|  | Female | 0.32 (0.28) | 0.39 (0.28) | - | - |
|  | Male | 0.45 (0.33) | 0.51 (0.32) | - | - |
| Coping | All | 0.47 (0.29) | 0.55 (0.28) | - | - |
|  | Female | 0.43 (0.27) | 0.5 (0.28) | - | - |
|  | Male | 0.52 (0.3) | 0.59 (0.28) | - | - |
| Pain | All | 0.65 (0.36) | 0.66 (0.35) | - | - |
|  | Female | 0.6 (0.36) | 0.61 (0.35) | - | - |
|  | Male | 0.7 (0.34) | 0.73 (0.35) | - | - |
| Senses | All | 0.86 (0.15) | 0.85 (0.16) | - | - |
|  | Female | 0.88 (0.13) | 0.87 (0.16) | - | - |
|  | Male | 0.86 (0.15) | 0.85 (0.16) | - | - |
| *Sense of belonging* | All | 44.7 (11.6) | 43 (10.5) | - | - |
|  | Female | 46 (11.4) | 44.4 (11.2) | - | - |
|  | Male | 43 (11.9) | 41.4 (9.4) | - | - |
| *Social support* | All | 9.3 (4.7) | 9.4 (4.6) | - | - |
|  | Female | 9.4 (4.9) | 9.3 (4.5) | - | - |
|  | Male | 9.4 (4.6) | 9.7 (4.7) | - | - |
| *Session density (%)* | All | - | 8 (5.2) | - | - |
|  | Female | - | 7.6 (4.8) | - | - |
|  | Male | - | 8.4 (5.7) | - | - |
| *Self-reported exercise (min/week)* | All | 121.4 (209) | 177.8 (201.3) | - | - |
|  | Female | 96.1 (175.5) | 154.9 (172.3) | - | - |
|  | Male | 143.9 (239.3) | 208.7 (232.4) | - | - |

| **Table S3: Outcome data for participants with at least T1-T3 data** | | | | | | |
| --- | --- | --- | --- | --- | --- | --- |
|  |  | **T1** | | **T2** | **T3** | **T4** |
| ***Sample sizes*** |  | ***n*** | | ***n*** | ***n*** | ***n*** |
|  | All | 82 | | 82 | 82 | - |
|  | Female | 44 | | 44 | 44 | - |
|  | Male | 38 | | 38 | 38 | - |
| ***Outcomes*** |  | ***Mean (SD)*** | | ***Mean (SD)*** | ***Mean (SD)*** | ***Mean (SD)*** |
| *Quality of Life* | | |  |  |  |  |
| Total utility score | All | 0.6 (0.22) | | 0.64 (0.21) | 0.65 (0.21) | - |
|  | Female | 0.58 (0.18) | | 0.61 (0.2) | 0.63 (0.21) | - |
|  | Male | 0.62 (0.26) | | 0.68 (0.22) | 0.67 (0.2) | - |
| Independent living | All | 0.73 (0.24) | | 0.76 (0.25) | 0.78 (0.25) | - |
|  | Female | 0.73 (0.22) | | 0.77 (0.25) | 0.75 (0.27) | - |
|  | Male | 0.74 (0.26) | | 0.76 (0.26) | 0.81 (0.23) | - |
| Relationships | All | 0.61 (0.32) | | 0.64 (0.3) | 0.65 (0.29) | - |
|  | Female | 0.62 (0.31) | | 0.65 (0.3) | 0.65 (0.3) | - |
|  | Male | 0.59 (0.33) | | 0.62 (0.3) | 0.66 (0.28) | - |
| Mental Health | All | 0.41 (0.32) | | 0.47 (0.32) | 0.46 (0.31) | - |
|  | Female | 0.38 (0.28) | | 0.41 (0.29) | 0.44 (0.33) | - |
|  | Male | 0.44 (0.35) | | 0.53 (0.34) | 0.49 (0.29) | - |
| Coping | All | 0.49 (0.3) | | 0.56 (0.29) | 0.58 (0.28) | - |
|  | Female | 0.47 (0.28) | | 0.52 (0.31) | 0.56 (0.3) | - |
|  | Male | 0.51 (0.33) | | 0.61 (0.26) | 0.61 (0.26) | - |
| Pain | All | 0.66 (0.36) | | 0.68 (0.36) | 0.67 (0.35) | - |
|  | Female | 0.62 (0.37) | | 0.6 (0.34) | 0.63 (0.35) | - |
|  | Male | 0.72 (0.35) | | 0.77 (0.35) | 0.72 (0.34) | - |
| Senses | All | 0.88 (0.14) | | 0.87 (0.17) | 0.89 (0.11) | - |
|  | Female | 0.89 (0.12) | | 0.89 (0.15) | 0.9 (0.11) | - |
|  | Male | 0.87 (0.15) | | 0.86 (0.18) | 0.89 (0.12) | - |
| *Sense of belonging* | All | 44 (11.2) | | 42.4 (9.8) | 42.6 (10.2) | - |
|  | Female | 44.5 (11.2) | | 42.9 (10.7) | 43.3 (10.7) | - |
|  | Male | 43.4 (11.3) | | 41.8 (8.7) | 41.8 (9.7) | - |
| *Social support* | All | 9.8 (4.5) | | 10.1 (4.9) | 9.9 (5) | - |
|  | Female | 10.1 (5) | | 10.1 (4.9) | 9.5 (4.8) | - |
|  | Male | 9.4 (3.9) | | 10.1 (5) | 10.4 (5.2) | - |
| *Session density (%)* | All | - | | 8.5 (5.3) | 5.5 (5.6) | - |
|  | Female | - | | 7.6 (4.4) | 5.4 (5.9) | - |
|  | Male | - | | 9.6 (6) | 5.5 (5.3) | - |
| *Self-reported exercise (min/week)* | All | 104.9 (148.5) | | 171.1 (185.2) | 190.7 (225.5) | - |
|  | Female | 100.8 (152.6) | | 164.6 (171.3) | 212.5 (259.7) | - |
|  | Male | 109.6 (145.4) | | 178.2 (201.6) | 164.7 (176.5) | - |

| **Table S4: Outcome data for participants with at least T1-T4 data** | | | | | |
| --- | --- | --- | --- | --- | --- |
|  |  | **T1** | **T2** | **T3** | **T4** |
| ***Sample sizes*** |  | ***n*** | ***n*** | ***n*** | ***n*** |
|  | All | 47 | 47 | 47 | 47 |
|  | Female | 24 | 24 | 24 | 24 |
|  | Male | 23 | 23 | 23 | 23 |
| ***Outcomes*** |  | ***Mean (SD)*** | ***Mean (SD)*** | ***Mean (SD)*** | ***Mean (SD)*** |
| *Quality of Life* | |  |  |  |  |
| Total utility score | All | 0.61 (0.22) | 0.65 (0.21) | 0.64 (0.21) | 0.66 (0.22) |
|  | Female | 0.55 (0.17) | 0.59 (0.18) | 0.6 (0.2) | 0.62 (0.21) |
|  | Male | 0.67 (0.24) | 0.71 (0.22) | 0.69 (0.22) | 0.7 (0.23) |
| Independent living | All | 0.72 (0.23) | 0.77 (0.25) | 0.77 (0.25) | 0.79 (0.22) |
|  | Female | 0.65 (0.23) | 0.74 (0.26) | 0.73 (0.27) | 0.75 (0.23) |
|  | Male | 0.79 (0.22) | 0.81 (0.24) | 0.82 (0.21) | 0.82 (0.2) |
| Relationships | All | 0.6 (0.32) | 0.65 (0.3) | 0.66 (0.29) | 0.67 (0.28) |
|  | Female | 0.58 (0.31) | 0.67 (0.3) | 0.69 (0.3) | 0.67 (0.27) |
|  | Male | 0.62 (0.35) | 0.63 (0.31) | 0.63 (0.29) | 0.66 (0.3) |
| Mental Health | All | 0.4 (0.31) | 0.46 (0.32) | 0.47 (0.32) | 0.49 (0.33) |
|  | Female | 0.3 (0.24) | 0.35 (0.26) | 0.4 (0.32) | 0.41 (0.29) |
|  | Male | 0.51 (0.34) | 0.57 (0.34) | 0.53 (0.3) | 0.58 (0.36) |
| Coping | All | 0.54 (0.32) | 0.57 (0.3) | 0.57 (0.26) | 0.58 (0.3) |
|  | Female | 0.49 (0.31) | 0.5 (0.33) | 0.52 (0.27) | 0.54 (0.29) |
|  | Male | 0.59 (0.32) | 0.64 (0.27) | 0.63 (0.25) | 0.62 (0.31) |
| Pain | All | 0.67 (0.35) | 0.69 (0.34) | 0.65 (0.36) | 0.67 (0.35) |
|  | Female | 0.54 (0.36) | 0.57 (0.33) | 0.55 (0.39) | 0.62 (0.36) |
|  | Male | 0.8 (0.3) | 0.81 (0.31) | 0.76 (0.29) | 0.73 (0.34) |
| Senses | All | 0.89 (0.13) | 0.89 (0.16) | 0.87 (0.12) | 0.9 (0.11) |
|  | Female | 0.9 (0.11) | 0.92 (0.1) | 0.88 (0.13) | 0.91 (0.1) |
|  | Male | 0.87 (0.15) | 0.85 (0.2) | 0.86 (0.12) | 0.9 (0.13) |
| *Sense of belonging* | All | 43.3 (10.9) | 41.8 (10.2) | 42.7 (10.3) | 41.9 (11) |
|  | Female | 44 (11.6) | 42.3 (11.4) | 43.6 (11.1) | 42.1 (10.6) |
|  | Male | 42.7 (10.3) | 41.2 (9) | 41.8 (9.5) | 41.7 (11.7) |
| *Social support* | All | 10 (4.7) | 10.4 (4.9) | 9.9 (4.2) | 10.2 (5.3) |
|  | Female | 10.9 (5.2) | 10.6 (5.1) | 9.5 (3.9) | 10.8 (5.2) |
|  | Male | 9.1 (4) | 10.3 (4.8) | 10.3 (4.5) | 9.5 (5.3) |
| *Session density (%)* | All | - | 9.4 (5.3) | 5.6 (5.9) | 6.9 (5.8) |
|  | Female | - | 8.2 (4.5) | 5.6 (5.5) | 6.4 (4.9) |
|  | Male | - | 10.7 (5.9) | 5.5 (6.3) | 7.4 (6.7) |
| *Self-reported exercise (min/week)* | All | 96.4 (127.4) | 165.8 (159.6) | 158.4 (206.7) | 290.7 (471.2) |
|  | Female | 86.7 (109.7) | 163.7 (145) | 198.8 (268) | 222.8 (238.7) |
|  | Male | 106.5 (145.4) | 167.8 (176.3) | 114.3 (95.1) | 358.5 (622.6) |
